# Analysis of Emotion and Recall in COVID-19 Advertisements: A Neuroscientific Study

**DOI:** 10.3390/ijerph18168721

**Published:** 2021-08-18

**Authors:** Miguel Baños-González, Mario Rajas-Fernández, Dolores Lucía Sutil-Martín

**Affiliations:** 1 Faculty of Communication Sciences, Rey Juan Carlos University, 28942 Fuenlabrada, Spain; miguel.banos@urjc.es; 2 Faculty of Law and Social Sciences, Rey Juan Carlos University, 28032 Vicálvaro, Spain

**Keywords:** neuroscience, advertisement, facial coding, emotion, recall, iMotions, AFFDEX

## Abstract

In this research, neuroscience techniques are applied to the field of marketing in the analysis of advertisements that include the COVID-19 pandemic in their stories. A study of emotion and memory in these audiovisual productions is carried out as two fundamental factors for the knowledge of consumer habits and decision making. By means of facial recognition biosensor systems (AFFDEX) and various tests, six informative and narrative, emotional and rational advertisements are presented to the subjects of the experiment to detect which emotions predominate; how they affect variables such as neuroticism, psychoticism or extroversion, among others; or what is remembered about the different works, brands and advertisers. Outstanding results are obtained in both emotional and cognitive analysis. Thus, in the field of public health, it is found that messages referring to COVID-19 included in advertisements are remembered more than other narratives or even the brands, products or services themselves. Likewise, joy is the predominant emotion, and its significance in such varied advertising stories stands out. Finally, it is clear that neuroscience research applied to marketing requires new methods and integrated applications to obtain satisfactory results in the advertising field.

## 1. Introduction

Applying neuroscience to the field of marketing allows us to address research in the field of advertising effectiveness, the study of consumers’ emotional responses or the analysis of brand, product or service recall [1]. Observing what reactions are produced in the consumer’s brain when understanding, getting excited about, or remembering an advertisement [2] is essential for defining communication strategies for advertisements aimed at decision-making [3,4].

So-called consumer neuroscience goes beyond the study of traditional conscious cognitive processes, which conventionally employ research methods and techniques typical of the social sciences, to enter the subject’s brain and analyse their unconscious cognitive processes [5]. The aim of neuroscience is to explain the preferences, motivations and expectations of the subject in order to predict his/her actions and, above all, to evaluate the success of advertising messages or stimuli from an innovative and effective methodological point of view that offers more satisfactory results than those obtained by traditional marketing test procedures [6].

Therefore, by applying advances in neuroscience to the specific field of marketing, emotions and feelings are placed at the centre of the analysis of consumer behaviour, incorporating perception, experience and memory as key elements [7], but, above all, placing in the foreground the relevance of the unconscious responses of subjects [8,9,10]. Doing so enables a better understanding of the effects of emotion on cognitive processes, such as perception, language, thought and memory, and consequently addresses three fundamental questions: the what, the how and the why of consumer behaviour when making purchasing decisions [11,12].

Cognitive neuroscience incorporates neuroimaging and biometric techniques to study in real time the processes that are triggered in the consumer’s brain and thus obtain objective, instantaneous and continuous data that allow relevant conclusions to be drawn. To achieve this, marketing neuroscience is based on models, methods, indicators, metrics, devices, tools and procedures that aim to measure, interpret and predict behaviour founded on direct emotional responses [13].

Specifically, to carry out research of this nature, different techniques are articulated to perform neuroscientific studies based mainly on functional magnetic resonance imaging (fMRI) [14,15,16], positron emission tomography (PET) [17], electroencephalography (EEG) [18], eye-tracking (ET) [19,20,21,22], galvanic skin response (GSR) [23] or, as far as this research is concerned, the analysis of facial micro-expressions [24].

Audio-visual forms of advertising provide important materials and stimuli for experiments in cognitive neuroscience applied to marketing. Provoking high levels of attention and emotion are sought in advertising messages because these cognitive responses are strongly correlated with recall and decision-making [25]. This is because advertisements with emotional content are more likely to be remembered than purely informative ones [26], since, having been emotionally involved, the viewer-consumer assimilates the message better by actively contributing to it with his or her different emotions.

The production of advertisements to be broadcast on television or on the Internet is closely linked to current events and to the social, cultural and economic transformations that are constantly taking place. For this reason, the advertising messages related to the COVID-19 pandemic we are currently experiencing provide a fertile field of research, mainly in the field of communication sciences applied to health.

In early 2020, the World Health Organisation (WHO) warned of a public health threat from a viral disease known as COVID-19 caused by the SARS-CoV-2 coronavirus. Subsequently, the WHO declared it a global pandemic.

The response by countries was based on establishing palliative and preventive measures to manage the disease. This pandemic not only endangers human life, but also endangers coexistence in general from an economic, political and social point of view [27]. Measures taken mainly focused on border closures, curfews and social distancing to reduce the spread of the virus, as well as the use of face masks and hydroalcoholic gel [28], while seeking to develop vaccines as the main remedy.

These changes in daily life generated uncertainty and caused stress, thus affecting mental health [29,30,31]. This resulted in emotional distress, social disorder, defensive responses and fear [32], among other pathologies.

Within this context, the psychological community considers personality as a fundamental factor affecting mental health. Personality therefore refers to an individual’s unique and stable way of thinking and acting and thus distinguishing themself from others. The evolution of personality theories from Eysenck’s three-factor theory [33], through Cattell’s sixteen-factor theory [34] or McCrae and Costa’s Big Five theory [35], including Tellegen and Waller’s seven-factor theory [36], indicate that emotion is a highly relevant component of personality.

Likewise, a large body of evidence indicates that personality largely explains and predicts emotions [37]. Some personality traits may be associated with negative emotions. For example, trait introversion is highly implicated with depression [38,39]. Similarly, extroversion and neuroticism cooperate as a mediating effect in the development of depression and anxiety. Specifically, research evidence [40,41] suggests that neuroticism is a potent mediator of anxiety.

This research analyses emotion and memory in a series of COVID-19-themed advertisements in this context of psychological hardship. Due to widespread vaccination, psychological hardship has subsided, but has not yet been overcome as the pandemic has not only affected health, but also the social and economic habits of citizens.

The main objective of this research article is to measure, analyse and evaluate emotion and memory in six advertisements referring to COVID-19 and to examine the explanatory power of other variables such as having suffered the disease.

The methods used are facial recognition with biosensors (AFFDEX) (Affectiva, Boston, MA, USA) and several specific tests to detect predominant emotions. The study evaluates the impact on emotional response of variables such as neuroticism, psychoticism or extroversion, and the memory of brands, products or services.

Relevant results are obtained in both the emotional and cognitive analyses. Among the main conclusions regarding public health, it was found that messages referring to COVID-19 are remembered more than other narrative moments or brands; likewise, joy is the predominant emotion triggered in the advertisements. Therefore, joy is the emotion that most influences decision making.

The results obtained allow us to conclude that messages related to the COVID-19 pandemic are remembered more than even well-known commercial brands. This finding poses a risk for highly visible brands that obtain high recall of the advertisement, but not of the commercial message they intend to convey to their target audience.

Finally, it was found that neuroscience applied to marketing requires new methods that provide relevant results in the field of advertising and health. This article shows that, using the tools of consumer neuroscience, it is possible to understand the emotions and recall of COVID-19 advertisements in the population, as well as their relationship with important variables such as having had the disease or whether a family member has been similarly affected.

Furthermore, in the field of facial recognition experiments with AFFDEX, this article includes interesting contributions both in the use of the device and the procedure carried out to obtain relevant results in the field of marketing.

## 2. Materials and Methods

The objectives of this research on advertising communication in the area of health are twofold. First, the study aims to measure and evaluate the basic emotions and recall of a series of advertising stimuli. Second, this analysis is supplemented through the consideration of variables such as having personally endured or having a close family member that has suffered the illness, gender, type of advertisement or levels of extroversion, neuroticism or psychoticism, to interpret the results of these health-related audio-visual productions.

The study involves quantitative-qualitative research that combines neuroscientific tools such as facial recognition or facial coding with social science techniques such as the completion of tests. This research analyses advertising effectiveness from two different perspectives: the application of neuroscientific techniques to marketing and traditional declarative techniques. This combination, especially demanded from the professional field, provides more complete results by analysing what happens in the brain when a subject is exposed to an advertising stimulus, and at the same time, to know what that individual remembers in relation to these stimuli. The recollection of the subject is what will ultimately allow us to assess whether the communication objectives set by the advertiser are achieved, and, if they are achieved, whether the effectiveness of the advertising action is confirmed.

In this way, research combining these two types of techniques contributes to the advancement and consolidation of a corpus that allows us to assess the capacity of neuromarketing to predict the effectiveness of an audiovisual advertisement by comparing the data obtained through neuroscience and those obtained from the responses of the subjects.

In this research, the neuroscientific technique of facial micro-expression analysis [42] was used, specifically applying Ekman and Friesen’s [43] Facial Action Coding System (FACS) model to the audio-visual advertising field.

FACS is the most widely used, comprehensive and rigorous facial coding system [44,45]. By recording subjects’ faces at the appropriate frame rate and playback speed, all possible facial expressions can be recorded and decomposed into so-called “Action Units” (AU). These AUs comprise the smallest facial movements that can be visually distinguished.

Historically, several FACS-based software packages have been used such as FaceReader-FEBE system (Noldus, Wageningen, Netherlands) [21], GfK-EMO Scan Software (GfK, Nuremberg, Germany) [46] and AFFDEX [47].

Research on facial expressions has been applied to research on how emotion virality occurs [48] or why some content is more easily shared than others [49].

In this study, the AFFDEX (Affectiva Emotion Analytics Dashboard) software module is used to record and analyse facial micro-expressions with the aim of capturing the subject’s levels of attention, basic emotions and participation when viewing certain audio-visual stimuli of an advertising nature on a screen.

AFFDEX is a cutting-edge tool, integrated in the iMotions software. By recording the subject’s face in front of a screen where the advertising stimulus is played, it allows the analysis of emotional responses in terms of Ekman’s basic emotions [50] and the interpretation of three other indicators: attention—based on the position of the head while the subject concentrates on receiving the stimuli; engagement—the emotional response triggered by the visual content; and valence—the positive or negative nature of the experience.

To date, AFFDEX has not been used frequently in the field of marketing, but several pioneering investigations have been carried out that have demonstrated its potential [51,52]. Similarly, in the field of health, it has been used in geriatrics [53], in forensics [54,55] and in pain studies [56].

The iMotions platform integrates AFFDEX within a set of techniques (EEG, EMG, GSR and ECG, among others) designed specifically for academic and professional research. Within iMotions 8.0, the Facial Expression Analysis Module was used to carry out the experiment considered in this research.

The module records the different subjects while they watch the advertisements and then uses indicators based on the biometric records captured by action units: 34 facial reference points (eyelids, eyebrows, nose, lips, jaw and so on), interocular distance or head position.

The raw values recorded by AFFDEX are transformed by the module in accordance with Ekman’s basic emotions. An indicator is added to each emotion that specifies the probability of its occurrence, establishing a range or scale of values from 0 to 100. AFFDEX recommends setting an arbitrary initial threshold of 50 to determine whether or not there is an emotional response [57]. In this research, records lower than this value are not included. Thus, for example, in Joy, records above 50 on this scale are taken into account and those below 50 are rejected.

The chosen population is made up of students between 18 and 25 years old, belonging to the demographic cohort “Generation Z” [58] residing in Madrid and having an active profile on a social network. The sample of 56 members (26 males and 30 females) was horizontal to create a bridge to new social networks, casting the sampling and recruitment net wider rather than deep. Therefore, the sampling used was horizontal networking by establishing a link to social networks, projecting a recruitment network breadth rather than depth [59].

The study sample was recruited through the following announcement on Twitter: “We are looking for students for a neuro-experiment on audio-visual advertising in the Brain Research Lab of URJC. It involves watching 6 spots on COVID-19 with eye-tracking and facial micro-expressions by @iMotionsGlobal”.

Given that children under the age of 18 need permission from their legal guardians to participate in this research and Generation Z is assumed to comprise people born between 1994 and 2010, it was decided that an age range between 18 and 25 would provide a representative sample. In addition, people in this cohort are generally more active on social media, tend to have multiple accounts, own smartphones, tend to follow fashion trends closely and are more permeable to unconscious perceptual processes [60].

Participants signed an informed consent form prior to participation. No personal data except for gender and age were used in this research. Subjects were coded as follows: 2021 + last 4 digits of their ID number.

For the selection of the audio-visual advertising productions that form part of the body of stimuli of this experiment, various models of analysis of television commercials have been used [61,62,63,64]. These studies coincide in pointing out that, in an advertisement, there is an integration of narrative messages—character, action, space and time—with the advertised content of the product or service itself, in such a way that the differential fact of a television advertising story lies in the way the two components are articulated. Therefore, for this experiment, we sought stimuli that highlight this dynamic relationship between what is advertised and what is narrated, between the advertising message and the rational or emotional content that constructs the audio-visual discourse.

The stimulus selection process consisted, in the first place, in pre-selecting 60 spots, 30 of a narrative and emotional nature produced by private advertisers and 30 of an informative and rational nature produced by public institutions or administrations, which met the following requirements: deal with COVID-19; have not been broadcast on mainstream television channels in Spain; have a duration of 0:50–1:20 min; and are works of manifest technical and creative quality following a specific evaluation model drawn up with professional criteria in which each particular advert had to score a minimum of 75 out of a possible 100 points.

In addition, a content analysis was carried out for each announcement to detect the following components: predominant emotion, salient narrative moments where a turning point or event of interest occurs, COVID-19 messages and significant changes in text, image or music.

Then, based on the aforementioned pre-selection, two focus groups were organised, each consisting of five experts in audio-visual advertising production, in order to make the final selection of the stimuli (Table 1). After viewing and discussion, the 6 pieces that form part of the research were chosen: 3 narrative advertisements that tell stories while advertising a product or service and 3 informative advertisements that describe a situation or offer information. The advertisements finally selected were:

The content of the six advertisements is very heterogeneous, with notably different usage of narrative and informative techniques and for the use of image and sound to convey messages and emotions.

“Thank You for not Riding” (Uber) is a “montage sequence” type advert: various characters perform everyday actions in an emotional atmosphere, until the (paradoxical) message from the advertiser is introduced: do not use Uber and stay at home doing quarantine.

“Not from Friends” (Poker): an advert for a beer brand using a humorous and cheerful story, in a musical genre, which conflicts with the seriousness of the COVID-19 pandemic.

#ABCD (Government of the Province of Buenos Aires) is an advertisement that introduces a version of the song “Aserejé” by the Spanish group “Las Ketchup” while repeating four COVID-19 hygiene and health recommendations for those using the Argentinean beaches.

Heineken’s “Connections” is another “montage sequence” type advert that illustrates everyday actions of video call connections while the characters consume the product.

“COVID-19 San Luis Campaign Vaccine” (Government of San Luis) is an advertisement that articulates real images with motion graphics animation and textual elements to illustrate the vaccination process.

“Mouthpieces” (Ministry of Public Health Uruguay) is a motion graphics animation advert that informs about different actions that must be taken to prevent contagion.

The experiment was conducted at the Brain Research Lab of the Universidad Rey Juan Carlos, Madrid, Spain, between 9 March 2021 and 17 March 2021.

A COVID-19 circuit was generated to carry out the experiment. The subject arrived in a room where the format of the experiment was explained, with the subject proceeding to provide its consent to participate. Subsequently, using the camera on their smartphone, they registered the QR code and accessed the EPQ-R Eysenck test to complete it.

For the examination of personality traits, we follow Eysenck [33], who proposed that the extraversion-introversion dimension (positive extraversion-affectivity marked by pronounced engagement with the external world and characterised by high sociability, talkativeness, energy and assertiveness) is caused by variability in cortical arousal.

With a low potential for environmental arousal, the cognitive performance of extraverts would be lower than that of introverts [65]. In addition to that, Eysenck’s model treats the dimensions of neuroticism and psychoticism as independent of extraversion. The model proposes that the stability dimension of neuroticism (neuroticism-negative affectivity marked by emotional instability and low tolerance to stress or aversive stimuli and characterised by anxiety, fear, moodiness, worry, envy, frustration, jealousy and loneliness) is explained by differences in the level of activity mainly in the limbic system. A relationship between unconscious processes, personality traits and decision-making can be found in many studies [66,67,68,69].

Once the test was completed, subjects were taken to another room in the Brain Research Lab, where the advertisements were viewed. Subjects entered the room and sat in front of a 14-inch monitor located at a distance of 50 cm on which the stimuli were projected, presented in a random order, which changed with each new subject to eliminate the possible influence of recency and primacy effects on the results obtained. At the top of the screen, there was a Logitech HD (Logitech, Lausanne, Switzerland) recording camera for visual recording.

Next, iMotions was calibrated to ensure that the facial micro-expression detection mask fully captured the subjects’ faces. Once iMotions achieved a 96% fit, the advertisements were viewed. The exposure of the advertisements was randomised. Each stimulus began with 1 s of blank screen. The room was kept at a constant temperature of 22 °C throughout the experimental phase.

The room was also isolated from the outside by a soundproofing system. We also used the same indirect lighting system for all participants, so the emotional comparison between groups of subjects was robust. The ventilation of the room was also maintained according to COVID-19 standards.

At the end of the viewing of the stimuli, the participants returned to the initial room to answer the questions related to the advertisements that made up the recall test.

In this sense, the analysis of advertisement recall carried out in different experiments [70] stands out as a fundamental technique to evaluate the outcome of an advertising message or stimulus and how affective messages lead to more immediate attitude changes, but cognitive adverts involve deeper processing and, therefore, achieve a greater retention effect in the subject [46].

In this test, each of the subjects must answer various questions related to the advertisements used as stimuli in the research; first, they are asked to remember both the brands and the different contents that appear in the advertisements: the activity of the advertisers, the message conveyed by each advertisement and the most significant sequences or images.

Subsequently, the subjects are asked questions related to the recall of the emotions they felt when faced with the stimuli: the advertisement(s) that moved them the most and an assessment, for each of the advertisements, of the intensity of the emotions, used as dependent variables in the research, using a 5-level Likert scale. Finally, they were asked which advertisements had the greatest impact or most captured their attention.

## 3. Results

### 3.1. Analysis of the Dominant Emotion

Table 2 shows the frequency of the expressions associated with each emotion in the participating subjects. With these data, we can identify, on the one hand, the dominant emotion in each of the advertisements that formed part of the sample and, on the other hand, the amount of emotion that each advertisement provoked as a proportion of the total number of expressions associated with emotions identified by the system.

In terms of the volume of emotional expressions, the most emotive advert was the one used by the Government of the Province of Buenos Aires, where 2.678 expressions were identified. This was closely followed by the Heineken advertisement with 2.373 expressions. At the opposite end of the scale is the Ministry of Public Health of Uruguay, where only 198 emotional expressions were identified, and the Government of San Luis, with 502 expressions (the two advertisements included in the sample in the rational category).

The dominant emotion was Joy in each of the advertisements used in this experiment. The case of Heineken stands out, with Joy accounting for almost 96% of the 2373 emotional expressions in the participating subjects. The Uber advert also showed Joy as responsible for close to 96% of the emotional expressions, but with a lower total number of expressions (1021). The advert for the Government of the Province of Buenos Aires invoked 2517 expressions of Joy, representing 94% of the total number of emotional expressions in that advert (2678).

A surprising finding of the investigation into advertisements related to the pandemic, which has caused countless family and personal dramas, is the low number of emotional expressions associated with Sadness in the subjects who participated in the experiment, representing only 1.87% of the total number of expressions identified in the six advertisements.

The Poker advert is the one that shows the greatest variety of emotional expressions. Despite the fact that happiness represents practically 60% of the identified expressions, Sadness represents around 12% of the expressions identified in the advert, Fear is shown in just over 9% of the expressions, Surprise in 8.4% and Anger in 7.3%. In this advert, situations are presented in which a group of young people, singing and dancing, show irresponsible attitudes to the global pandemic, with the video ending with the logo of the beer brand, which may be surprising for the market segment to which the company that signs the message belongs.

### 3.2. Influence of Variables on Emotions

In this section, we observe the influence of each of the independent variables analysed in the experiment on the dependent variable (Anger, Disgust, Fear, Joy, Surprise and Sadness) using the ANOVA model (analysis of variance); in other words, we check whether factors such as the type of advertisement (emotional or rational), gender, whether the subject has had COVID, and the subject’s extroversion contribute to the variance of each emotion, which will allow us to determine which of the study variables have acted as predictors of any of the emotions.

Next, we analyse the effect of the factors on the different emotions investigated (dependent variables); these factors are:

- Emo_Rat: type of advert, emotional or rational;
- Gender: gender of the subject of the experiment;
- COVID_Suj: if the subject has suffered the disease or not;
- COVID_Fam: whether or not a family member of the subject has had the disease;
- Extroversion: arousal levels;
- Neuroticism: emotional instability;
- Psychoticism: lack of control and adaptability;
- L_Scale: level of sincerity of the subject.

#### 3.2.1. Influence of Variables on Emotion Anger

Table 3 shows that only four factors of the model have a significant influence on this dependent variable; in this case, having had COVID, extroversion, neuroticism and psychoticism contribute to the variance of Anger (significance ≤ 0.05). In the last three factors (extroversion, neuroticism and psychoticism) the influence on the dependent variable Anger is negative; on the contrary, the fact of having or not having had the disease contributes positively to the variance of Anger.

It is also seen that whether the advertisements are emotional or rational, the gender of the subject, whether a family member of the participant has had COVID and the level of sincerity do not have a significant impact on the dependent variable Anger (significance ≥ 0.005).

#### 3.2.2. Influence of Variables on Emotion Disgust

In relation to the dependent variable Disgust, as shown in Table 4, we observe that there are only two factors with a significant influence on this emotion: extroversion and a family member experiencing COVID (significance ≤ 0.05). In both cases, the influence on the dependent variable Disgust is negative.

According to these data, it can be affirmed that variables such as gender, the relationship with COVID of the subjects, neuroticism, psychoticism or the level of sincerity do not have the ability to significantly predict the dependent variable Disgust, since the significance value is greater than 0.05 in all cases.

#### 3.2.3. Influence of Variables on Emotion Fear

In the case of the dependent variable Fear, there are only two factors with a significance level of less than 0.05 (Table 5); the fact that the message is rational or emotional (significance = 0.000) and that a relative of the respondent has had the disease (significance = 0.000) have a significant influence on the dependent variable Fear. However, in the first case (Emo_Rat), the influence is positive, while in the second (COVID_Fam), the influence is negative.

The other variables (gender, whether the subject has suffered COVID, extroversion, neuroticism, psychoticism and the subject’s level of sincerity) do not have a significant influence on the variance of the dependent variable Fear.

#### 3.2.4. Influence of Variables on Emotion Joy

Table 6 describes the effect of each of the factors analysed on the dependent variable Joy. In this case, only one factor, extroversion, has no significant effect on this dependent variable (significance = 0.684).

For all other factors investigated, the influence on Joy is significant (significance ≤ 0.05). In the case of whether the advertisement is rational or emotional, gender, whether or not the subject has had the illness, neuroticism and psychoticism, the influence on Joy is negative. Only two factors contribute positively to the variance of Joy: having a relative who has had the illness and the level of sincerity.

#### 3.2.5. Influence of Variables on Emotion Surprise

For the dependent variable Surprise (Table 7), only two of the factors in the model have a significant effect on this emotion: gender (significance = 0.002) and the subject’s level of sincerity (significance = 0.034). In both cases, the influence on Surprise is positive.

The other factors analysed (whether the advertisement is rational or emotional, whether the subject or a family member has had the illness, extroversion, neuroticism and psychoticism) do not show a significant influence on Surprise.

#### 3.2.6. Influence of Variables on Emotion Sadness

For the dependent variable Sadness (Table 8), only neuroticism has a significant effect on this emotion (significance = 0.005); in this case, the influence on Sadness is negative. The rest of the factors analysed (whether the advertisement is rational or emotional, gender, whether the subject or one of their relatives has had the illness, extroversion, psychoticism and level of sincerity) do not show a significant effect on the dependent variable Sadness.

### 3.3. Memory Analysis

The results of the questionnaire designed to measure the recall of the subjects who participated in the experiment show important differences between one advertisement and another. It should be recalled that a sample of advertisements was selected that presents an important variety of possibilities both in the form and content of the messages.

We assess recall in relation to 2 advertisements for well-known brands (Uber and Heineken) versus 4 for brands and institutions unknown in the context in which the experiment was carried out. In turn, among the unknown brands, there are two adverts identified by the experts as emotional (Poker and Government of the Province of Buenos Aires) and two others considered rational (Government of San Luis and Ministry of Public Health of Uruguay).

Among the well-known brands, there is one message (Uber) in which the brand has a very limited presence, appearing only once, at the end of the advert, and its content can be considered paradoxical, as the images show the opposite of what the public might expect from a company dedicated to passenger transport: people locked in their homes and two pieces of advice, stay at home and do not travel. The Heineken advert, on the other hand, resorts to images of product use, with a strong brand presence, in contexts where beer is consumed, albeit in virtual meetings.

Including the little-known brand advertisements, we have: Poker beer, which shows images of young people who irresponsibly dance and sing without masks during a pandemic, with the brand appearing in the final shot; the Government of the Province of Buenos Aires, which uses images of the beach and holidays together with basic rules to avoid contagion, all sung to the rhythm of “Aserejé”; the advert of the Government of San Luis, which describes the process to get vaccinated in this city; and finally, the Ministry of Public Health of Uruguay, who explains, through animations, the importance of using masks.

When asked which brands they remember (Table 9), the majority of subjects remembered Heineken (43) and Uber (39). The fact that the most remembered brands are the most well-known is an expected result; however, there is very little distance between the two well-known brands despite the big differences in the advertisements: in the case of Uber, there is nothing related to the use of the service, and the brand appears only at the end, while the brand presence in Heineken is very frequent, and different situations of the use of the product being consumed are shown almost continuously.

In relation to the lesser-known brands, two advertisements considered emotional are the most remembered by the subjects who participated in the experiment: Government of the Province of Buenos Aires (20) and Poker (16). However, 13 subjects remember an advertisement that we can describe as rational (it describes the process that the population of San Luis must follow to get vaccinated) from an institution unknown in the environment of the participants in the experiment, namely the Government of San Luis; the cause of this level of recall, which we can consider as unexpected, may be due to the fact that the subjects also remember having felt Joy when they saw the advertisement, mainly because of the arrival of the vaccine. Finally, no one remembers the mark corresponding to the other rational message, with recommendations to avoid infection with the coronavirus, signed by the Ministry of Public Health of Uruguay. Only one person claimed not to remember any of the brand names they had seen in the experiment.

In terms of advertiser activity, the situation is slightly different with respect to brand recall:

- Beer/drink: 46.
- Transport: 32, although sometimes identified with taxi service, cars, etc.
- Vaccine: 9.
- Tourism: 8.

In addition, regardless of whether subjects recall the advertisers’ activity, some subjects consider that what is being advertised are measures against the coronavirus, without specifying whether they refer to vaccines, the use of masks or maintaining a responsible attitude towards the pandemic.

As we can see, beer and the passenger transport company occupy the first two positions; however, the vaccine is remembered more than what the Government of the Province of Buenos Aires advertises (tourism).

If we relate the recollection of the activity carried out by the advertisers with the recall of the message conveyed by the advertisements, we see that, in general, the subjects recall messages related to the coronavirus and the pandemic, regardless of the brand or the institution that signs the advertisement. The importance of wearing a mask, staying at home, enjoying oneself while respecting safety measures, the importance of vaccination, appeals to individual and group responsibility and the risks of not following the rules in the face of COVID are some of the most recurrent messages identified by the subjects who participated in the experiment.

In the case of beer brands and Uber, there is no perceived message associated with a commercial objective of the brand in the ads; generally, a message related to the fight against the coronavirus is captured: the best thing to do in the current situation is to stay at home (Uber), enjoy from a distance (Heineken) or if we do not act with caution the situation will not improve (Poker). The most attractive and impactful advertisements for the subjects who participated in the experiment are those with emotional components (Table 10):

The order is the same as in the case of brand recall, with the two least attractive advertisements to the subjects (Government of San Luis and Ministry of Public Health of Uruguay) having the lowest levels of brand recall.

Finally, subjects were also asked about the emotion they remembered feeling when watching each of the advertisements. At this point, the advertisements for which the subjects recall having felt the most emotion are the ones that obtain the highest levels of recall; this is so regardless of the previous knowledge that the subjects have of that brand. Thus, a brand unknown in the environment of the interviewees (Poker) is remembered by 16 participants, who say they feel Anger and Surprise when they see the advert: Anger because of the irresponsibility of the situations presented and Surprise because, in the end, it is signed by a brand of beer.

Something similar happens with the announcement of the Government of the Province of Buenos Aires, which, for the interviewees, is the one that has made them feel the most Joy, recalling the institution that signs the message 20 of the participants.

A special case is that of the Uber brand, as the participants felt, among other emotions, a significant level of Surprise to see that the brand that signed an advertisement so focused on confinement, and that asks us not to leave home, is the one that provides passenger transport services.

At the other extreme, the subjects do not remember feeling any special emotion with the advertisement whose content was limited to explaining measures against the coronavirus, and none of them remember the institution signing the advertisement: the Uruguayan Ministry of Public Health.

If we compare the recall of the emotion that the subjects have felt before a given advertisement, and the dominant emotion in each advertisement that we obtained from the table of frequencies elaborated from the data of the Facial Coding software, we can observe relevant coincidences and differences. To measure the recall of the emotion that the subjects claim to have felt before an advertisement, a scale with five levels was used, where 1 means not having felt that emotion and 5 means having felt that emotion in a very intense way. As we have seen in the methodology, the subjects filled in the questionnaire to recall a certain time after having finished watching the advertisements.

Facial Coding measures the automatic and unconscious response of the subjects to a stimulus (in this experiment, a series of advertising messages) while measuring the subjects’ memory of the emotion they have felt. A declarative technique is used where the response is elaborated by the participants with time to think about what they have felt during the advertisement. Therefore, we assess affective cognitive congruence in terms of the level of coincidence between the subjects’ answers (cognitive response obtained through the questionnaire) and the data obtained with the Facial Coding software (affective response of the subjects).

Despite the differences shown by the tools used to obtain the data, the advertisement of the Government of the Province of Buenos Aires is the one in which the greatest number of expressions associated with Joy has been identified, data obtained with Facial Coding (2517 expressions out of a total of 2678), and the one in which the subjects remember feeling more Joy based on the data obtained from the questionnaire (4.08 out of 5). The data also coincide in the Heineken advert, the subjects’ recollection of Joy (3.96 out of 5) reflecting the information obtained with Facial Coding (2270 out of 2373 expressions).

One advert that shows important differences is the Poker advert. According to the data obtained during the viewing of the message, happiness represents almost 59.92% of the emotional expressions. However, the subjects claim to have felt Surprise (3.23 out of 5), Anger (2.80) and Sadness (2.45), with happiness being relegated to fourth place (2.25 out of 5). The subjects justify their Surprise by the brand behind the message and by the attitude of the young people, namely their Anger at their irresponsibility and behaviour.

The biggest differences in the data obtained are in the Uber advert, where the emotion that the subjects remember feeling the most is Sadness, while only four expressions associated with this emotion were recorded during the viewing of the advert. Participants felt Sadness because they remembered situations experienced during the pandemic, because of the images of the elderly, because they were unable to leave the house or because they were unable to have contact with other people.

## 4. Discussion

In examining the connections between the observed results, there is a clear interrelationship between the frequency of expressions associated with emotions, the variables that influence these emotions and recall.

In this sense, in the case of Joy, the predominant emotion in the six advertisements, it is also the one that is affected by the greatest number of variables, and only extroversion, of the eight analysed, does not have a significant influence on this emotion. Likewise, as has been shown, it is one of the most frequently mentioned emotions and a determining factor in the recall of the material seen. Thus, in the advertisements for the Government of the Province of Buenos Aires and Heineken, the highest number of expressions of Joy were recorded (2517 and 2270 expressions, respectively) and, at the same time, it was in those where the subjects recalled having felt the most Joy (4.08 and 3.96 out of 5). In the same vein, Sadness, Anger and Disgust, the least frequent emotions in the different advertisements analysed, are less influenced by the variables incorporated and are significantly less remembered.

Comparing the results with other experiments that have used neuromarketing techniques [51,52] and recall tests [46,70], this research confirms that the most consistent messages from an emotional point of view are also the most remembered and those that can provoke, in that sense, changes in the subject’s attitude.

With respect to personality traits (extroversion, psychoticism and neuroticism), which remain stable over time in individuals [28], the analysis of the data indicates that these traits negatively affect the different emotions after viewing the advertisements. Anger is influenced by extroversion, psychoticism and neuroticism, respectively. That is, a high level of extroversion means that the person’s experience of Anger is reduced. The same is true for the other two personality traits. It has also been shown that for Joy, the personality traits that have a negative influence are psychoticism and neuroticism. That is, people with a high level of both psychoticism and neuroticism show less Joy. However, the personality trait of extroversion negatively influences Disgust while neuroticism negatively influences Sadness.

These results are in line with those obtained in other studies [71,72] where personality traits are related to emotions, as described by Heffner et al. [73] in a similar research on the influence of emotions on COVID-19 related advertising.

This research confirmed that the most emotional advertisements are also the most remembered by the subjects. The data obtained in this experiment confirm the conclusions presented in different studies that have used a wide range of research techniques, both neuroscience and declarative [26,70].

The limitations of this work are noted. First, the sample of advertisements, being very heterogeneous, may have conditioned certain results in the frequency of micro-expressions of emotions, the dependent variables analysed or recall (by incorporating known brands together with other unknown brands).

Second, the current phase of the COVID-19 pandemic, in which a large part of the population is being vaccinated and a certain general optimism is perceived, may have influenced the low values of emotions such as Sadness, Anger and Fear, which would probably have reached higher levels in other stages of the complicated situation experienced in 2020 and the beginning of 2021 due to the spread of the virus.

Similarly, the age of the subjects in the experiment (18–24) may have been an important factor in the under-representation of some emotions.

## 5. Conclusions

Cognitive neuroscientific techniques based on the recording of reactions to stimuli by means of biosensors in conjunction with declarative methods such as the recall test make it possible to obtain conclusions regarding research on audio-visual advertising, such as the one presented in this article.

First of all, the subjects of the experiment remember messages related to the COVID-19 pandemic more than narrative actions, commercial messages and even brands or institutions in advertisements.

This fact is very significant for the production of advertisements: On the one hand, associating a product or service with certain social or health areas can provide added value for advertisers, as well as generating important messages for the public, such as those related to health. However, according to the data obtained in this research, this association can also entail risks, as the subjects do not always perceive the brand message by remembering only the health or social message.

With regard to the search for effectiveness in the emotional construction of advertisements, Joy (by far) is the emotion that provokes the most reactions or responses and, therefore, manages to involve the viewer more in the reception of the visual and sound stimuli of the advertisement. By involving the viewer, in addition to demanding a more proactive viewing on their part, it can lead to a change in behaviour or decision making.

The importance of incorporating study variables such as neuroticism, psychoticism or extroversion into the neuroscientific study has been confirmed. These are indicators that provide relevant data for the study of the brain of the subjects of the experiment and, as reflected in the data from this research, may have a relevant role in the effects caused by audio-visual commercial messages.

None of the independent variables analysed significantly influence all the dependent variables (the different emotions). Only three dependent variables are significantly affected by extroversion (Anger, Disgust and Sadness) and a family member of the subject having suffered COVID (Disgust, Fear and Joy). Each of the other factors only significantly influence two emotions, which allows us to conclude that emotions are affected by very different factors and that not all of them influence emotions in the same way.

In the same sense, when processing the information at a cognitive level, using the declarative technique of the recall test, the subject remembers the advertisement that has moved them most, although they do not remember the exact emotion. In other words, they recognise having felt an emotion, although the subject is not able to really identify the emotion they have felt, which is why it differs from the AFFDEX recording of the micro-expressions associated with that emotion. In relation to this point, it was found that when the subjects declare having felt an intense emotion (whether or not this coincides with the micro-expressions), brand recall is very high; thus, in the case of brands unknown in the environment of the interviewees, the advertisements remembered by the subjects as more emotional obtain much higher levels of recall than those obtained with advertisements which, according to the recall data of the subjects, transmit very low levels of emotion.

Although it is not possible to conclude the influence of the context or the social moment on the emotions shown by the subjects, it is interesting to see that, in analysing the emotions generated by advertisements with content related to the pandemic, the subjects hardly show emotions such as Sadness or Fear, despite the dramatic situations that have been experienced since the pandemic began. It seems appropriate to interpret that the progress of vaccination and the fact that the protective measures were producing results have had a notable influence on the subjects’ emotions.

Similarly, it is interesting to note that recall tests, as a cognitive tool, have an outstanding potential to complement results such as those obtained in the face recognition experiments in an important phase such as that of overcoming the COVID-19 pandemic and the invention of vaccines.

For this reason, it is necessary to continue to conduct experiments that analyse informative and emotional advertisements in different geographical and temporal contexts and, using the tools of neuroscience, to achieve satisfactory results in the field of marketing communications.

## Figures and Tables

**Table 1:** List of advertisements.

Title	Advertiser	YouTube link	Country	Duration
Thank you for not Riding	Uber	https://www.youtube.com/watch?v=_e8XLnMiCOE (accessed on 7 February 2021)	USA	1:15
Not from Friends	Poker	https://www.youtube.com/watch?v=I_dKjwZEwWA (accessed on 5 February 2021)	Colombia	1:17
#ABCD	Government of the Province of Buenos Aires	https://www.youtube.com/watch?v=8AuOBanae_w (accessed on 30 January 2021)	Argentina	0:59
Connections	Heineken	https://www.youtube.com/watch?v=WZnHkv5-z4k (accessed on 1 February 2021)	USA	1:07
COVID-19 San Luis Campaign Vaccine	Government of San Luis	https://www.youtube.com/watch?v=9jbLwbPUMTM (accessed on 10 February 2021)	Argentina	0:58
Mouthpieces	Ministry of Public Health Uruguay	https://www.youtube.com/watch?v=bH0EljQvZgc (accessed on 15 January 2021)	Uruguay	0:51

**Table 2:** Frequency of expressions associated with each emotion.

	Poker		Bs. As.		Uber		Heineken		San Luis		Uruguay		TOTAL	
Excitement	F	%	F	%	F	%	F	%	F	%	F	%	F	%
**Anger**	53	7.30	17	0.63	6	0.56	4	0.17	32	6.37	9	4.55	121	1.60
**Disgust**	25	3.44	24	0.90	18	1.69	50	2.11	26	5.18	1	0.51	144	1.91
**Fear**	66	9.09	37	1.38	13	1.22	20	0.84	34	6.77	2	1.01	172	2.28
**Joy**	435	59.92	2517	93.99	1021	95.69	2270	95.66	327	65.14	147	74.24	6717	89.04
**Sadness**	86	11.85	22	0.82	4	0.37	17	0.72	0	0.00	12	6.06	141	1.87
**Surprise**	61	8.40	61	2.28	5	0.47	12	0.51	83	16.53	27	13.64	249	3.30
**Total**	726	100	2678	100	1067	100	2373	100	502	100	198	100	7544	100

**Table 3:** Analysis of variance for the dependent variable Anger. Parameter estimates.

Parameter	B	Standard Error	t	Sig.	95% Confidence Interval	
					Lower Limit	Upper Limit
Interception	91.315	14.369	6.355	0.000	62.844	119.786
Emo_Rat = 0.00	−5.776	3.160	−1.828	0.070	−12.037	0.485
Emo_Rat = 1.00	0 ^a^					
Gender = 000	5.408	12.718	0.425	0.671	−19.791	30.607
Gender = 1.00	0 ^a^					
COVID = 0.00	38.211	13.642	2.801	0.006	11.181	65.240
COVID = 1.00	0 ^a^					
COVID_Fam = 0.00	7.660	5.558	1.378	0.171	−3.352	18.673
COVID_Fam = 1.00	0 ^a^					
Extroversion	−0.575	0.159	−3.605	0.000	−0.891	−0.259
Neuroticism	−0.285	0.133	−2.136	0.035	−0.549	−0.021
Psychoticism	−0.418	0.133	−3.156	0.002	−0.681	−0.156
L_Scale	0.180	0.137	1.316	0.191	−0.091	0.450

a. This parameter is set to zero because it is redundant.

**Table 4:** Analysis of variance for the dependent variable *Disgust*. Parameter estimates.

Parameter	B	Standard Error	t	Sig.	95% Confidence Interval	
					Lower Limit	Upper Limit
Interception	83.700	34.711	2.411	0.017	15.029	152.371
Emo_Rat = 0.00	30.847	21.129	1.460	0.147	−10.954	72.647
Emo_Rat = 1.00	0 ^a^					
Gender = 0.00	17.998	25.135	0.716	0.475	−31.728	67.724
Gender = 1.00	0 ^a^					
COVID = 0.00	39.808	27.483	1.448	0.150	−14.563	94.179
COVID = 1.00	0 ^a^					
COVID_Fam = 0.00	−12.835	5.302	−2.421	0.017	−23.325	−2.345
COVID_Fam = 1.00	0 ^a^					
Extroversion	-0.483	0.239	−2.024	0.045	-0.956	-0.011
Neuroticism	-0.026	0.140	-0.182	0.856	-0.303	0.252
Psychoticism	0.099	0.120	0.826	0.410	-0.138	0.335
L_Scale	-0.059	0.168	-0.350	0.727	-0.392	0.274

a. This parameter is set to zero because it is redundant

**Table 5:** Analysis of variance for the dependent variable Fear. Parameter estimates.

Parameter	B	Standard Error	t	Sig.	95% Confidence Interval	
					Lower Limit	Upper Limit
Interception	93.686	8.336	11.239	0.000	77.225	110.146
Emo_Rat = 0.00	10.334	1.712	6.037	0.000	6.954	13.714
Emo_Rat = 1.00	0 ^a^					
Gender = 0.00	−8.605	5.913	−1.455	0.148	−20.282	3.072
Gender = 1.00	0 ^a^					
COVID = 0.00	4.851	5.072	0.956	0.340	−5.164	14.865
COVID = 1.00	0 ^a^					
COVID_Fam = 0.00	−15.247	3.794	−4.019	0.000	−22.738	−7.756
COVID_Fam = 1.00	0 ^a^					
Extroversion	−0.093	0.102	−0.913	0.362	−0.294	0.108
Neuroticism	−0.157	0.085	-1.851	0.066	−0.325	0.010
Psychoticism	−0.163	0.086	−1.905	0.059	−0.332	0.006
L_Scale	−0.168	0.123	−1.368	0.173	−0.410	0.075

a. This parameter is set to zero because it is redundant.

**Table 6:** Analysis of variance for the dependent variable Joy. Parameter estimates.

Parameter	B	Standard Error	t	Sig.	95% Confidence Interval	
					Lower Limit	Upper Limit
Interception	100.131	0.954	105.010	0.000	98.261	102.000
Emo_Rat = 0.00	−1.946	0.861	−2.261	0.024	−3.634	−0.259
Emo_Rat = 1.00	0 ^a^					
Gender = 0.00	−12.459	3.735	−3.336	0.001	−19.781	−5.137
Gender = 1.00	0 ^a^					
COVID = 0.00	−6.952	0.671	−10.366	0.000	−8.267	−5.637
COVID = 1.00	0 ^a^					
COVID_Fam = 0.00	1.198	0.467	2.563	0.010	0.282	2.114
COVID_Fam = 1.00	0 ^a^					
Extroversion	−0.004	0.009	−0.406	0.684	−0.021	0.014
Neuroticism	−0.034	0.008	−4.478	0.000	−0.049	−0.019
Psychoticism	−0.039	0.008	−4.874	0.000	−0.055	−0.023
L_Scale	0.037	0.008	4.343	0.000	0.020	0.054

a. This parameter is set to zero because it is redundant.

**Table 7:** Analysis of variance for the dependent variable Surprise. Parameter estimates.

Parameter	B	Standard Error	t	Sig.	95% Confidence Interval	
					Lower Limit	Upper Limit
Interception	54.002	19.226	2.809	0.005	16.123	91.880
Emo_Rat = 0.00	5.926	9.111	0.650	0.516	−12.023	23.875
Emo_Rat = 1.00	0 ^a^					
Gender = 0.00	19.331	6.022	3.210	0.002	7.466	31.195
Gender = 1.00	0 ^a^					
COVID = 0.00	16.446	8.403	1.957	0.052	−0.110	33.001
COVID = 1.00	0 ^a^					
COVID_Fam = 0.00	−9.806	17.043	−0.575	0.566	−43.383	23.772
COVID_Fam = 1.00	0 ^a^					
Extroversion	−0.025	0.130	−0.194	0.846	−0.282	0.231
Neuroticism	−0.150	0.155	−0.967	0.335	−0.455	0.155
Psychoticism	0.042	0.148	0.285	0.776	−0.250	0.335
L_Scale	0.195	0.091	2.136	0.034	0.015	0.374

a. This parameter is set to zero because it is redundant.

**Table 8:** Analysis of variance for the dependent variable Sadness. Parameter estimates.

Parameter	B	Standard Error	t	Sig.	95% Confidence Interval	
					Lower Limit	Upper Limit
Interception	73.570	33.702	2.183	0.031	6.899	140.241
Emo_Rat = 0.00	−2.636	3.559	−0.741	0.460	−9.677	4.405
Emo_Rat = 1.00	0 ^a^					
Gender = 0.00	−0.863	32.284	−0.027	0.979	−64.729	63.003
Gender = 1.00	0 ^a^					
COVID = 0.00	−0.253	14.656	−0.017	0.986	−29.246	28.740
COVID = 1.00	0 ^a^					
COVID_Fam = 0.00	−11.351	13.181	−0.861	0.391	−37.426	14.724
COVID_Fam = 1.00	0 ^a^					
Extroversion	−0.331	0.452	−0.732	0.466	−1.224	0.563
Neuroticism	−0.257	0.090	−2.848	0.005	−0.436	−0.079
Psychoticism	0.443	0.540	0.820	0.414	−0.626	1.512
L_Scale	0.291	0.425	0.684	0.495	−0.550	1.132

a. This parameter is set to zero because it is redundant.

**Table 9:** Brand recall.

Brand	Recall (Subjects)
Heineken	43
Uber	39
Government of the Province of Buenos Aires	20
Poker	16
Government of San Luis	13
Ministry of Public Health of Uruguay	0
No recollection of marks	1

**Table 10:** Advertisements considered most appealing and impactful.

Brand	Recall (Subjects)
Heineken	43
Uber	39
Government of the Province of Buenos Aires	20
Poker	16
Government of San Luis	13
Ministry of Public Health of Uruguay	0
No recollection of marks	1

## Data Availability

Data sharing not applicable.
